# Treatment of iron deficiency and iron deficiency anemia with intravenous ferric carboxymaltose in pregnancy

**DOI:** 10.1007/s00404-018-4782-9

**Published:** 2018-05-08

**Authors:** Bernd Froessler, Tijana Gajic, Gustaaf Dekker, Nicolette A. Hodyl

**Affiliations:** 1 Department of Anesthesia, Lyell McEwin Hospital, Haydown Road, Elizabeth Vale, SA 5112 Australia; 2 Discipline of Acute Care Medicine, University of Adelaide, Adelaide, SA 5005 Australia; 3 Flinders Medical Centre, Bedford Park, SA 5042 Australia; 4 Department of Obstetrics and Gynecology, Lyell McEwin Hospital, Elizabeth Vale, SA 5112 Australia; 5 The Robinson Research Institute, Adelaide Medical School, University of Adelaide, Adelaide, SA 5006 Australia

**Keywords:** Pregnancy, Iron deficiency no anemia, Iron deficiency anemia severity, Intravenous iron, Ferric carboxymaltose, Safety

## Abstract

**Purpose:**

To evaluate the efficacy and safety of intravenous ferric carboxymaltose administration to pregnant women with varying severities of iron deficiency anemia and iron deficiency without anemia.

**Methods:**

In this prospective observational study of local obstetric practice, we analyzed data from 863 pregnant women with iron deficiency according to anemia status and severity. All women were treated with intravenous ferric carboxymaltose in pregnancy. Treatment efficacy was assessed by repeat hemoglobin measurements at 3 and 6 week post-infusion and ferritin levels, where available. Safety was assessed by analysis of adverse events, fetal heart rate monitoring, and newborn health outcome data.

**Results:**

Ferric carboxymaltose significantly increased hemoglobin in women with mild, moderate, and severe iron deficiency anemia and women with iron deficiency alone at 3 and 6 week post-infusion (*p* < 0.01 for all). No hemoconcentration occurred in iron-deficient women without anemia. No serious adverse events were recorded, with minor temporary side effects (including local skin irritation, nausea, and headache) occurring in 96 (11%) women. No adverse fetal or neonatal outcomes were observed.

**Conclusions:**

Ferric carboxymaltose infusion corrects iron deficiency or various degrees of iron deficiency anemia efficaciously and safely pregnant women, and does not cause hemoconcentration.

## Introduction

Iron deficiency (ID) and iron deficiency anemia (IDA) in pregnancy are global health issues, affecting around 30% of women in high-resourced countries, and increasing to over 50% of women in low-resourced countries [1]. It is well recognized in that both conditions are associated with adverse physiological and psychological outcomes in mother and child. For the mother, these include cardiovascular problems, reduced physical activity and cognitive performance, reduced immune function, tiredness, and increased depressive episodes, while, for the infant, these include preterm birth, fetal growth restriction, intrauterine fetal death, low Apgar scores, and neonatal infection [2–6].

Uncertainty remains whether non-anemic iron-deficient pregnant women require iron replacement and whether intravenous (IV) iron should be considered or could potentially be concerning by causing hemoconcentration [5]. In other patient groups, iron-deficient non-anemic (IDNA) individuals were found to have worse mental, physical health outcomes, executive functioning (EF), which were discharged later from hospital after surgery and had higher mortality rates at 90 days after hospital discharge [7–9].

In addition, women entering labor in an anemic state have a reduced ability to compensate for peri-partum hemorrhage and are, therefore, at increased risk for morbidity and mortality [10]. This risk can become amplified in the context of caesarean section, given the increased blood loss that occurs with this intervention [11]. Anemia also increases the need for a peri-partum allogeneic red blood cell (RBC) transfusion, which is independently associated with increased morbidity [11, 12]. While RBC transfusion often remains the default treatment option [13], it only corrects hemoglobin temporarily and not the underlying condition [14, 15].

Iron stores are not routinely assessed in the antenatal setting and iron supplementation is often only initiated when anemia is detected. The adequate assessment and treatment of ID and IDA during pregnancy, as part of routine antenatal care, may, therefore, be beneficial for both maternal and newborn health [16]. Different levels of anemia, patients’ response, compliance, and tolerability to oral iron require individualized treatment. Oral iron replacement is often considered as the first-line treatment [17]. However, it can be ineffective, cause intolerable adverse events, or may not facilitate urgent rapid iron repletion [18, 19]. Intravenous iron administration is an alternative treatment option for IDA in pregnancy and has been recommended in various guidelines [17]. The uptake of this administration route has been hindered by perceived barriers and misconceptions, discussed elsewhere [20–22], and has led to the development of new, safer types of iron formulation. Data from observational studies on the efficacy and safety of these new treatments are critical to guide clinical management decisions, and to assure the safety of expecting mothers and the unborn fetus. Christoph et al. demonstrated a comparable safety profile to iron sucrose for ferric carboxymaltose (FCM) in a retrospective analysis [23]. Our group also reported the safe and effective use of FCM in the treatment of ID/IDA in the second and third trimesters of pregnancy in a small observational study (*n* = 65) [21] whether women with ID, mild, moderate, and severe anemia tolerate this treatment, and whether it is successful in the correction of a low hemoglobin in women not responding to oral iron or too late in gestation. However, data on FCM administration during pregnancy remain limited and have been published only from six studies in a total of 634 women [17]. In addition, there is a little knowledge about the erythropoietic response to IV iron in pregnant women with IDNA and IV iron administration appears to be a concern for many clinicians [22]. Therefore, we now assess the efficacy and safety of this treatment in the so far largest single cohort, a further 863 pregnant women, according to the baseline severity of ID/IDA, to enhance general knowledge and to examine the effect on pregnant women with IDNA.

## Methods

This retrospective study was approved by the Queen Elizabeth Hospital, Lyell McEwin Hospital, and Modbury Hospital Human Research and Ethics committee (Reference number 2011160). Consent from individual participants was not required, because all the measurements and demographic information were collected as part of routine care. All women were referred for IV iron administration by the obstetric team for a peri-partum iron infusion as part of individual antenatal care. Risks and benefits were discussed as part of routine clinical management. The most recent hemoglobin and in most cases ferritin results from routine antenatal visits, prompted the decision, mostly since these women were too close to term to opt for oral iron, were not responding to oral iron or not compliant with oral iron treatment.

FCM is the institutional IV iron formulation of choice, and as per hospital protocol, women were prescribed up to a maximum of 20 mg of FCM per kg bodyweight. The vast majority of women received 1000 mg of FCM.

Available data from women who received FCM infusions as outpatients in the Women’s Assessment Unit at the Lyell McEwin Hospital (Elizabeth Vale, South Australia) between August 2012 and December 2016 were analyzed.

All blood samples were collected prior to infusion and then again, where clinically indicated, at up to two post-infusion visits (at approximately 3 and 6 weeks). Hemoglobin and ferritin concentrations were determined in the hospital laboratory using sodium lauryl sulphate (SLS) method for Hb analysis (Sysmex XE2100 analyzer) and direct chemiluminometric sandwich immunoassay (Siemens ADVIA Centaur XP) for ferritin analysis. Women were observed for 60 min following the infusion, before being discharged home. Medical and pathology data were collated from case notes and electronic laboratory reports, as well as transfusion data linkage reports.

### Statistical analysis

For analysis, women were subdivided based on their hemoglobin concentrations prior to the iron infusion, with severe anemia defined as < 90 g/L, moderate anemia defined as 90–94 g/L, mild anemia defined as 95–110 g/L, and iron deficiency with no anemia defined as > 110 g/L. ANOVA or Kruskal–Wallis tests were used to compare demographic data and baseline hemoglobin and ferritin data between the four ID and anemia groups, using Bonferroni post hoc comparisons (or Mann–Whitney *U* tests applying a Bonferroni correction) where indicated. Changes in hemoglobin over the pre- and post-infusion periods were analyzed comparing across the four groups using repeated-measures ANOVA, with time (pre-infusion, 3 and 6 weeks) as the repeated measure. Due to the loss of follow-up data at the 6 week time point and to maximize the power of the analysis, two separate repeated-measures ANOVAs were performed—the first comparing between pre-infusion and 3 weeks and the second comparing between 3 and 6 weeks. To adjust for multiple comparisons, an a priori adjustment was made to the critical *p* value using the Bonferroni method for this analysis Frequency data (including side effect rates) were analyzed using the Chi-squared test. All analyses were conducted using SPSS (v24). *p* values < 0.05 were considered to indicate statistical significance.

## Results

The demographic characteristics of the 863 women receiving FCM for iron deficiency or iron deficiency anemia are presented in Table 1. At the time of the infusion, women were defined as having mild anemia (*n* = 462; 54%), while 88 (10%) had moderate anemia, 79 (9%) had severe anemia, and 234 (27%) had iron deficiency with no anemia. Most women in this study were Caucasian, with a mean BMI in the overweight range. Women with ID and severe anemia were significantly younger (*p* = 0.01) and had a significantly lower BMI than women with ID and no anemia (*p* < 0.01; Table 1). Most women gave birth by vaginal delivery. Less than one-third of women in each anemia severity group were taking oral iron supplements prior to infusion (Table 1). No differences were observed in hematological characteristics (hemoglobin and ferritin) at the booking appointment conducted at 12 week gestation (Table 2).

**Table 1:** Demographic and clinical characteristics of women according to anemia status at infusion

	ID no anemia (*n* = 234)	ID mild anemia (*n* = 462)	ID moderate anemia (*n* = 88)	ID severe anemia (*n* = 79)	*p*
Age (years)	28 ± 6	28 ± 6	26 ± 6	26 ± 6^a^	0.01
BMI	29 ± 8	27 ± 8	26 ± 5	25 ± 5^a^	< 0.01
Gravidity	3 (2–4)	3 (2–4)	3 (2–4)	3 (2–5)	0.84
Parity	1 (1–2)	1 (1–3)	1 (1–2)	1 (0–3)	0.54
Mode of delivery					
Vaginal	120 (51%)	260 (56%)	55 (62.5%)	51 (64.5%)	0.50
Elective caesarean	57 (24%)	92 (20%)	9 (10%)	17 (21.5%)	
Emergency caesarean	31 (13%)	60 (13%)	11 (12.5%)	4 (5%)	
Instrumental/episiotomy	20 (9%)	42 (9%)	12 (14%)	4 (5%)	
Unknown	6 (3%)	8 (2%)	1 (1%)	3 (4%)	
Pre-infusion supplements					
Oral iron	30 (13%)	94 (20%)	24 (27%)	26 (33%)	0.65
Oral pregnancy formula (contains iron)	19 (8%)	42 (9%)	12 (14%)	9 (11%)	0.38
Gestational diabetes	47 (20%)	61 (13%)	9 (10%)	10 (13%)	0.29
Blood loss (estimated) (mL)	350 (250–500)	300 (200–500)	300 (200–500)	300 (200–500)	0.48
Gestational age at intervention (weeks)	35 (32–37)	35 (32–37)	35 (32–37)	35 (30–36)	0.13

Data are presented as mean ± SD, median (interquartile range), or *n* (%)

^a^*p* < 0.05 (post hoc comparison) ID severe anemia compared to ID no anemia

**Table 2:** Haematological characteristics of women according to anemia status at infusion

	ID no anemia (*n* = 234)	ID mild anemia (*n* = 462)	ID moderate anemia (*n* = 88)	ID severe anemia (*n* = 79)	*p*
Hemoglobin at booking (12 weeks) (g/L)	124 (118–131)	115 (107–123)^a^	113 (103–122)^a^	106 (92–119)^a^	< 0.01
Ferritin at booking (12 weeks) (µg/L)	21 (10–38)	16 (8–34)	12 (6–24)	8 (5–23)	0.23

Data are presented as median (interquartile range)

^a^*p* < 0.05 (post hoc comparison) in each group compared to the ID with no anemia group

**Fig. 1:**
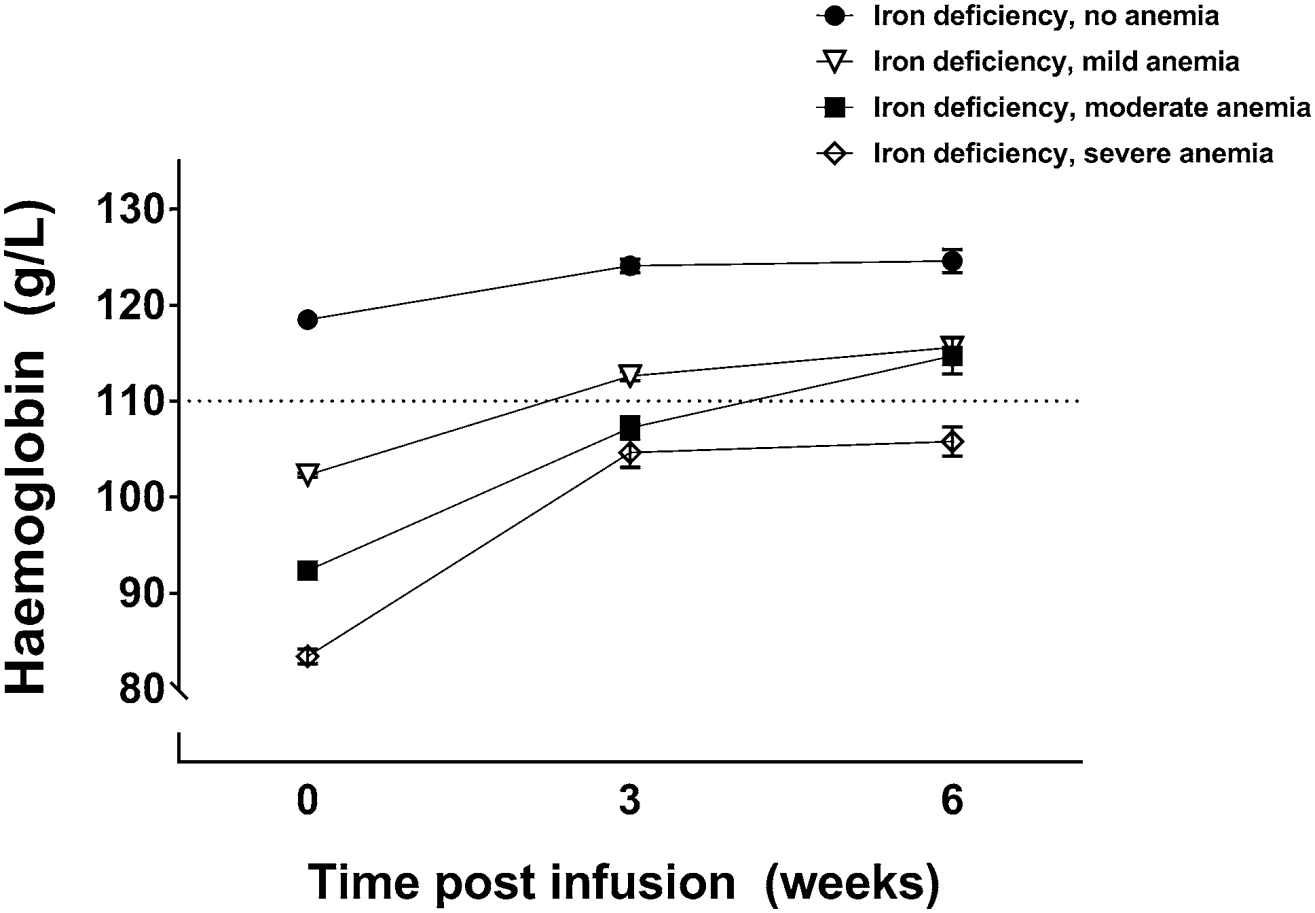
Hemoglobin levels (mean ± SEM) across the testing period according to the severity of iron deficiency anemia prior to infusion. Dotted line reflects adequate hemoglobin concentration in second and third trimester of pregnancy (110 g/L). No anemia (n = 234), mild anemia 96–110g/L (n = 462), moderate anemia 90–95/L (n = 88), severe anemia < 90 g/L (n = 79)

Following the infusion, samples were collected as required by the obstetric team for determination of hemoglobin values; data were available for 84% of the women at visit 1 (3 week post-infusion) and 34% of the women at visit 2 (6 week post-infusion). All women had at least one control visit. Changes in hemoglobin concentrations over the post-infusion period are presented in Fig. 1.

Hemoglobin levels collected at the first post-infusion time point (3 weeks) were significantly higher in all severity groups compared to pre-infusion levels (0 weeks) (*p* < 0.001 for all severity groups). At the first post-infusion time point (3 weeks), hemoglobin had significantly increased by an average (± SEM) of 5.1 (± 1.3) g/L in women with ID and no anemia, 9.8 (± 0.6) g/L in women with ID mild anemia, 15.3 (± 1.2) g/L in women with ID moderate anemia, and 21.5 (± 1.5) g/L in women with ID severe anemia. At 3 week post-infusion, hemoglobin concentrations were above the accepted adequate level for pregnant women (110 g/L) in only the ID no anemia and the ID mild anemia groups (Fig. 1).

Hemoglobin concentrations did not significantly change between the first and second post-infusion visits (at 3 and 6 weeks) in both women with ID no anemia, and in women with ID and severe anemia (Fig. 1). However, in women with ID and both mild and moderate anemia, hemoglobin was significantly higher at the second compared to the first post-infusion visit (*p* < 0.01 for both). In women with mild anemia, the average (± SEM) increase between 3 and 6 week post-infusion was 6.8 (1.2) g/L, while, in the moderate anemia group, the increase was 14.2 (4.1) g/L. At 6 week post-infusion, hemoglobin concentrations were above the recommended adequate level for pregnant women (110 g/L) in all women except the ID severe anemia group (Fig. 1).

Ferritin values were available for a subset of women at each time visit and in the post-partum period. Ferritin significantly decreased between the booking and infusion visits (*p* < 0.001; Table 3). Ferritin levels at 3 week post-infusion were significantly higher than pre-infusion levels (*p* < 0.001) and remained elevated at 6 week post-infusion in the subset of women assessed (*n* = 28). In the subset of women who had ferritin measured post-partum (*n* = 27), levels were very low (Table 3).

**Table 3:** Ferritin levels (µg/L) across the testing period

	Booking	Pre-infusion	3 week post-infusion	6 week post-infusion	Post-partum
Ferritin (μg/L)	16 (8–34) *n* = 514	7 (5–10) *n* = 725	188 (111–284) *n* = 106	137 (46–256) *n* = 28	17 (11–23) *n* = 27

Data are presented as median (IQR)

Adverse reactions are presented in Table 4. Minor adverse events occurred in 96 (11%) of women overall, with only 31 (3.5%) women reporting more than one adverse effect. The most common adverse event was local irritation/pain of the skin at the site of infusion, which occurred in 29 women (3%), while each of the other adverse effects was observed in less than 3% of women (Table 4). The frequency of adverse events differed significantly between the severity groups (*p* = 0.029), with the lowest number of adverse events observed in the no anemia group (*n* = 16, 7%) and the highest rates observed in the mild (*n* = 54, 12%) and moderate anemia groups (*n* = 18, 20%). Fetal heart rate monitoring did not indicate a drug-related adverse effect on the fetal heart rate pattern.

**Table 4:** Adverse events in all women combined and according to severity of anemia

Adverse events	All women (*n* = 863)	ID no anemia (*n* = 234)	ID mild anemia (*n* = 462)	ID moderate anemia (*n* = 88)	ID severe anemia (*n* = 79)
Local (irritation/pain)	36 (4%)	4 (2%)	26 (6%)	3 3(%)	3 (4%)
Headache/dizziness	29 (3%)	6 (3%)	12 (3%)	10 (11%)	1 (1%)
Vascular (hypotension)	26 (3%)	5 (2%)	14 (3%)	5 (6%)	2 (2.5%)
Nausea/vomiting	21 (2%)	4 (2%)	11 (2%)	5 (6%)	1 (1%)
Musculoskeletal	6 (1%)	0 (0%)	4 (1%)	2 (2%)	0 (0%)
Respiratory	9 (1%)	2 (1%)	4 (1%)	1 (1%)	2 (2.5%)
Total number of patients reporting any adverse event	96 (11%)	16 (7%)	54 (12%)	18 (20%)	8 (10%)
Patients reporting more than one adverse event	31 (3.5%)	5 (2%)	17 (4%)	8 (9%)	1 (1%)

Data are presented as *n* (% of women in same group)

Neonatal characteristics of the study population are presented in Table 5. Infants born to mothers with ID and moderate anemia had a significantly greater birth weight than those born to women without anemia (*p* < 0.01). All other neonatal anthropomorphic characteristics of the anemia groups did not differ significantly from each other. Apgar scores were similar across all anemia severities.

**Table 5:** Neonatal characteristics of the whole cohort (*n* = 863) and according to anemia status at infusion

	All births (*n* = 863)	ID no anemia (*n* = 234)	ID mild anemia (*n* = 462)	ID moderate anemia (*n* = 88)	ID severe anemia (*n* = 79)	*p* ^a^
Birth weight (g)	3454 ± 545	3432 ± 536	3468 ± 496	3581 ± 596^b^	3308 ± 726	0.025
Length (cm)	50 ± 2	50 ± 2	50 ± 2	50 ± 2	49 ± 3	0.146
Head circumference (cm)	35 ± 2	35 ± 2	35 ± 2	35 ± 2	35 ± 2	0.672
Apgar 1 min	9 (8–9)	9 (8–9)	9 (8–9)	9 (8–9)	9 (8–9)	0.324
Apgar < 7	62 (7%)	15 (6%)	34 (7%)	8 (9%)	5 (6%)	0.895
Apgar 5 min	9 (9–9)	9 (9–9)	9 (9–9)	9 (9–9)	9 (9–9)	0.379
Apgar < 7	8 (1%)	3 (1%)	4 (1%)	1 (1%)	0 (0%)	0.345

Data presented as mean ± SD, median (IQR) or *n* (%)

^a^Main effect of anemia severity groups

^b^*p* < 0.01 versus no anemia (post hoc analysis)

## Discussion

This large case series of 863 pregnant women strengthens the evidence on the safety and efficacy of IV iron administration with FCM in pregnancy in women. This includes 234 women who were iron-deficient and not anemic. No serious adverse events occurred amongst our patients, with only 11% of women experiencing mild adverse events, which were mostly self-limiting.

ID or IDA are very common conditions, with estimates that over 40% of women globally are affected [24]. ID and IDA increase maternal mortality and morbidity, particularly in the context of peri-partum hemorrhage, with rates of preterm delivery and low birth weight also increased [3]. While oral iron supplementation can increase hemoglobin and ferritin levels in pregnancy, we have previously demonstrated the safe and effective use of FCM infusion in pregnant women during the second and third trimesters of pregnancy [21]. Importantly, we also highlighted improved quality of life in these women, which has ongoing benefits to mental health and adoption of health-promoting behaviors [25]. We now expand this work to assess safety and efficacy of a single FCM infusion in women with differing severities of IDA and IDNA. This large cohort study demonstrates effective restoration of hemoglobin levels for up to 6 week post-infusion, regardless of anemia severity, with no serious adverse outcomes noted. The rate of minor adverse events was like that reported previously and was not amplified in any of the groups studied this time.

IDA has been the third leading cause globally for years lived with disability (YLDs) since 1990 and remained in this position over the last 2 decades [26]. In addition, it is ranked 13 for disability-adjusted life-years (DALYs) [27]. The impact on mental health, cognitive function, physical performance, work capacity, and general health is a substantial burden for the affected individual and society [9, 28, 29]. Personal and economic loss is enormous but preventable and treatable [30]. Progression to IDA poses additional risks and increases women’s vulnerability; particularly should a peri-partum hemorrhage occur. Transfusion of blood products in the obstetric setting is not a rare event, varying between 1.6 and 3% [12]. In addition, the incidence of peri-partum hemorrhage is on the rise and has increased by 33% between 2001 and 2010 [12]. Worldwide bleeding at childbirth remains the most common cause for maternal death [31]. Correcting hemoglobin by RBC transfusion remains a temporary measure if the underlying condition is not treated [32]. Optimizing iron stores prior to elective surgery associated with an increased risk of bleeding has been part of the wider concept of Patient Blood Management (PBM) and has been recommended in a consensus statement by international experts [33]. Women booked for elective caesarean section or entering labor with an increased risk of bleeding fall into the same category and deserve, therefore, to be managed accordingly [16, 17, 34].

The significance and risk of ID for the child is well described. By treating ID in pregnancy, birthweight improves, and short and long-term consequences of ID can be prevented [3, 4, 35]. In the current cohort, the average birth weight was comparable to population norms (mean 3355 g) [36]. Birth weight was not different between infants whose mothers had mild, moderate, and severe anemia, however, which was higher in the moderate anemia group compared to women with no anemia. While this was observed and the magnitude birthweight difference within a healthy weight range (approximately 150 g) is clinically definitely significant, it is unlikely to be a direct consequence of FCM infusion, given that all women in the current study received this treatment, with no difference observed between the iron deficiency no anemia group compared to both the mild and severe groups.

The cycle from persistent ID in pregnancy to residual post-partum ID occurs in many women and can be compounded by heavy menstrual bleeding [25]. Fatigue is one of the most common presentations in the post-partum period, associated with low concentration, low mood, depressive symptoms, and irritability [29]. All will impact on ability to care for the newborn [37–39]. This raises the question for the ideal treatment approach for women with iron deficiency and emphasizes the importance of follow-up and potential ongoing treatment in the post-partum period. Interestingly, six women from our previous publication [21] were in this new cohort in their consecutive pregnancy where they again presented with profound ID or IDA. Despite intravenous iron replacement therapy in their previous pregnancy, these women were unable to sustain normal iron stores into and during their next pregnancy. Addressing ID in pregnancy and beyond, therefore, becomes a crucial component of antenatal and postnatal care and appears to gain wider acceptance amongst clinicians [22].

A limitation of our study is the lack of a control group and the retrospective nature of the data. The strength, however, is that this large cohort reflects clinical practice of a large obstetric unit at a university teaching hospital in metropolitan setting, and shows clear benefit of this therapy in improving iron stores in women with ID.

In conclusion, our results strengthen the evidence that IV iron in the form of FCM is safe and efficacious to treat IDA and IDNA in pregnancy. The timely detection and appropriate treatment of ID can significantly improve maternal health and pregnancy outcomes and may offer a circuit breaker for women with one of the most common diseases globally.
